# Supplementary material for: NaCl-Templated Ultrathin 2D-Yttria Nanosheets Supported Pt Nanoparticles for Enhancing CO Oxidation Reaction
Source: Nanomaterials (Basel). 2022 Jul 5;12(13):2306. doi: 10.3390/nano12132306 (PMC9268161; doi:10.3390/nano12132306)
Supplement: Supplementary file 1 [file nanomaterials-12-02306-s001.zip › nanomaterials-1792678-supplementary.pdf]

nanomaterials

Supplementary Information

NaCl-Templated Ultrathin 2D-Yttria Nanosheets Supported Pt Nanoparticles for Enhancing CO Oxidation Reaction

Luozhen Jiang †, Chen Tian †, Yunan Li, Rui Si, Meng Du, Xiuhong Li *, Lingling Guo * and Lina Li *

Shanghai Advanced Research Institute, Chinese Academy of Sciences, Shanghai 201210, China; lzjiang@mail.ustc.edu.cn (L.J.); tianchen@sinap.ac.cn (C.T.); liyunan@sinap.ac.cn (Y.L.); sirui@mail.sysu.edu.cn (R.S.); dum@sari.ac.cn (M.D.)

* Correspondence: lixiuhong@zjlab.org.cn (X.L.); guolingling@zjlab.org.cn (L.G.); lilina@sinap.ac.cn (L.L.)

† These authors contributed equally to this work.

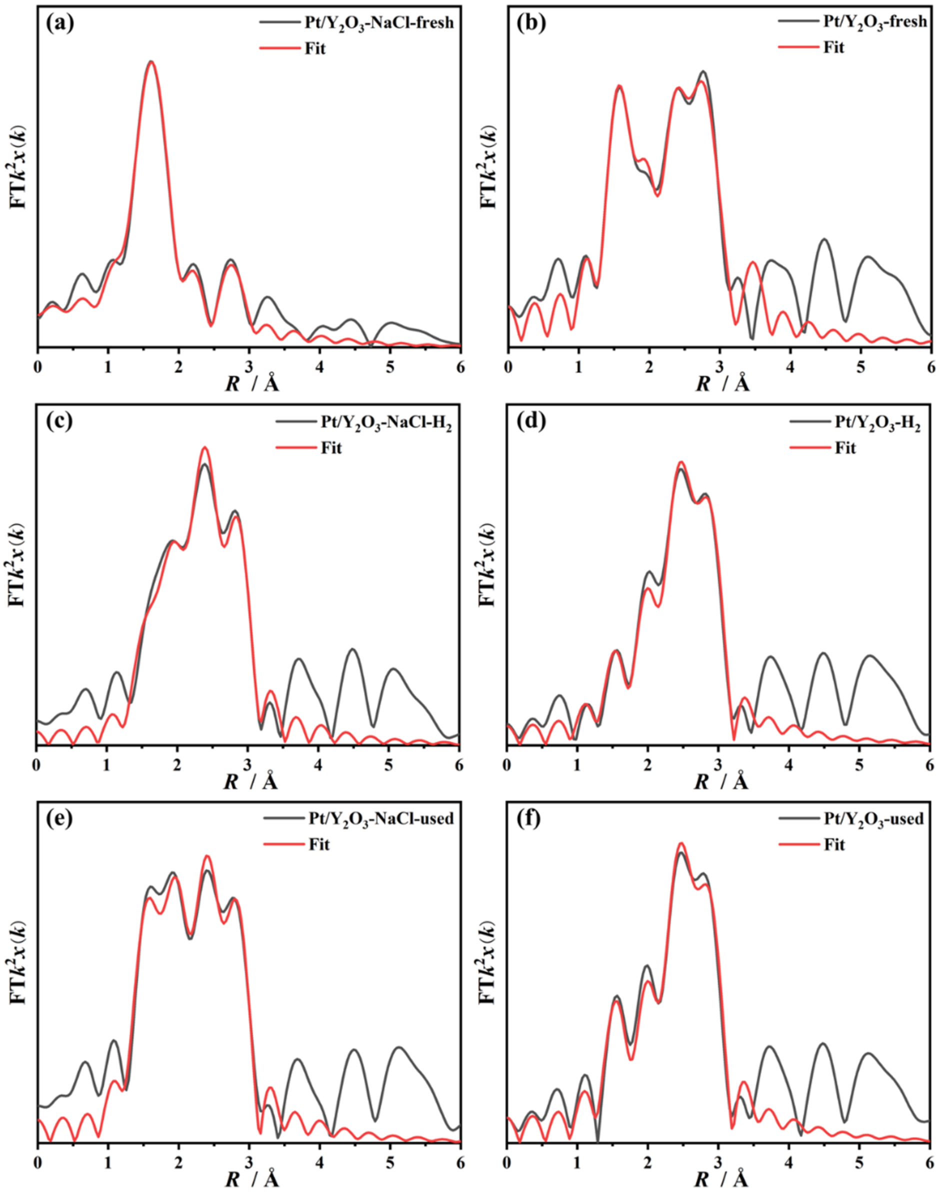

Figure S1. EXAFS fitting curves in R space of Pt/Y_2O_3 and Pt/Y_2O_3-NaCl catalysts.

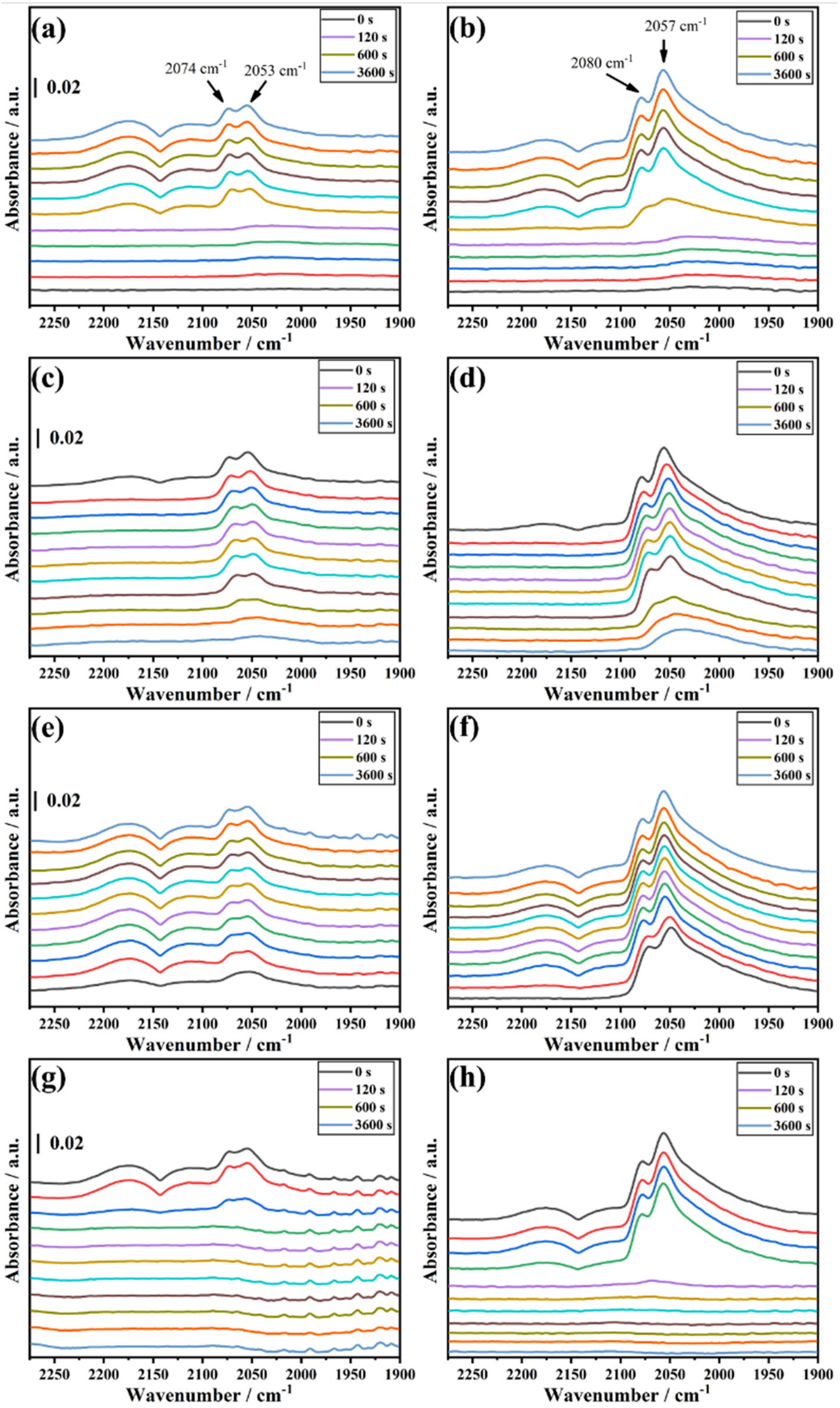

Figure S2. *In situ* DRIFTS of Pt/Y_2O_3 **(a,c,e,g)** and Pt/Y_2O_3-NaCl **(b,d,f,h)** catalysts during CO adsorption−desorption: (a,b) CO adsorption; (c,d) N_2 purging; (e,f) CO resorption; (g,h) O_2 removal.
